# WGT: Tools and algorithms for recognizing, visualizing, and generating Wheeler graphs

**DOI:** 10.1016/j.isci.2023.107402

**Published:** 2023-07-14

**Authors:** Kuan-Hao Chao, Pei-Wei Chen, Sanjit A. Seshia, Ben Langmead

**Affiliations:** 1 Department of Computer Science, Johns Hopkins University, Baltimore, MD 21218, USA; 2 Department of Electrical Engineering and Computer Sciences, University of California, Berkeley, Berkeley, CA 94720, USA

**Keywords:** Bioinformatics, Algorithms, Data structure

## Abstract

A Wheeler graph represents a collection of strings in a way that is particularly easy to index and query. Such a graph is a practical choice for representing a graph-shaped pangenome, and it is the foundation for current graph-based pangenome indexes. However, there are no practical tools to visualize or to check graphs that may have the Wheeler properties. Here, we present Wheelie, an algorithm that combines a *renaming heuristic* with a permutation solver (Wheelie-PR) or a Satisfiability Modulo Theory (SMT) solver (Wheelie-SMT) to check whether a given graph has the Wheeler properties, a problem that is NP-complete in general. Wheelie can check a variety of random and real-world graphs in far less time than any algorithm proposed to date. It can check a graph with 1,000s of nodes in seconds. We implement these algorithms together with complementary visualization tools in the WGT toolkit, available as open source software at https://github.com/Kuanhao-Chao/Wheeler_Graph_Toolkit.

## Highlights

• Wheeler graphs are the basis of many pangenome and other sequence analysis tools
• The WGT generates, recognizes, and visualizes Wheeler graphs
• WGT’s Wheelie algorithm efficiently distinguishes Wheeler and non-Wheeler graphs
• WGT’s bipartite Wheeler graph visualizer illustrates Wheeler graphs orderings

## Graphical abstract

**Figure:**
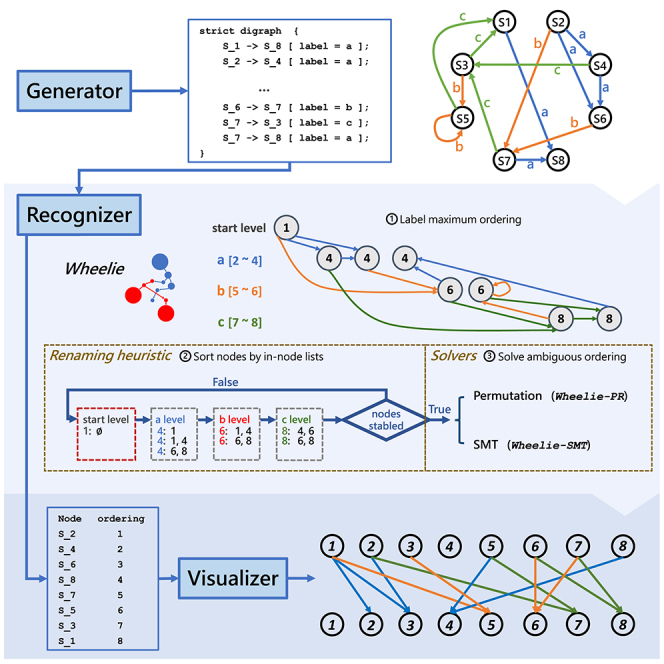

## Introduction

A Wheeler graph is a class of directed, edge-labeled graph that is particularly easy to index and query. It is a generalization of the Burrows-Wheeler-Transform (BWT)-based FM index,^1^ and partly forms the basis for existing pangenome alignment tools such as vg.^2^^,^^3^

A graph is a Wheeler graph when its nodes can be totally ordered according to the co-lexicographical order of the sets of strings spelled out on all paths leading into the nodes. Formally: an edge-labeled, directed graph is a Wheeler graph if and only if there exists a total ordering over its nodes such that 0-indegree nodes come before all other nodes in the ordering, and for all pairs of edges, (u,v) and $\left(u^{\prime },v^{\prime }\right)$ labeled *a* and $a^{\prime }$, respectively: (i) $a≺a^{\prime }\to v<v^{\prime }$, and (ii) $a=a^{\prime }\wedge u<u^{\prime }\to v\leq v^{\prime }$.

Many graph- and tree-shaped structures relevant to genomics either have the Wheeler graph properties or can be modified to have them. These include certain trees (via the XBWT),^4^ De Bruijn graphs,^5^ and reverse deterministic graphs derived from multiple alignments.^6^ The discovery of this unified definition of Wheeler graphs will help researchers to develop still more BWT variants, e.g., for pangenome indexing. For instance, the GCSA study proposed building a reverse deterministic graph from a multiple alignment, then modifying the graph through a repeated path doubling procedure, ultimately yielding a Wheeler graph.^6^ This approach can generate smaller graphs while preserving genome coordinates, compared to the more widely used De Bruijn graph, as demonstrated in Figure S3.

Despite the utility of the the Wheeler graphs mentioned previously,^2^^,^^6^^,^^7^^,^^8^ there are no tools or libraries that make it easy to use Wheeler graphs or to check if a particular graph has the requisite properties. This problem is NP-complete in general and hard to approximate.^9^ An exponential-time algorithm was proposed by Gibney & Thanckachan,^9^ but no implementation is available.

We present Wheeler graph toolkit (WGT), an open source suite for generating, recognizing, and visualizing Wheeler graphs. WGT includes functionality for generating graphs that do or do not have the Wheeler properties. Two generators produce De Bruijn graphs and tries derived from one or more input sequences provided as FASTA. Another generator produces reverse deterministic graphs^6^ from multiple sequence alignments. A fourth generator produces random graphs parameterized by the desired number of nodes, edges, distinct edge labels (i.e., alphabet size), and the most number of outgoing same-label edges.

Central to WGT is the fast Wheelie algorithm for Wheeler graph recognition. The algorithm combines a *renaming heuristic* with two alternate solvers, both capable of reaching exact solutions to the recognition problem. One solver uses an exhaustive search over possible node permutations, and the other uses a Satisfiability Modulo Theory (SMT) solver.^10^ We call the overall algorithm “Wheelie”, while we use the names “Wheelie-Pr” and “Wheelie-SMT” for the versions that use the permutation and SMT solvers, respectively. When run on a Wheeler graph, Wheelie also reports a node ordering for which the properties are satisfied and indexes the graph into *O*, *I*, and *L* three bitarrays,^11^ which are useful inputs to a downstream tool for pattern matching.

Here, we benchmark Wheelie’s solvers in comparison to each other and to the algorithm proposed by Gibney and Thankachan.^9^ We benchmark with a variety of input graphs, including graphs derived from real multiple alignments of DNA and protein sequences. We also use randomly generated graphs with various configurable characteristics. Finally, we implement and demonstrate a visualizer that allows the user to picture the graph in light of the Wheeler properties.

In the following, *G* denotes a directed graph, *N* its set of nodes, and *E* its set of edges, with n=|N| and e=|E|. Σ denotes the set of edge labels appearing on at least one edge, with σ=|Σ|.

## Results

Graphs used for evaluation were generated using WGT’s generator algorithms, which can produce (a) De Bruijn graphs, (b) tries, (c) a reverse deterministic graphs derived from a multiple alignments, (d) *complete* random Wheeler graphs, and (e) a *d-NFA* random Wheeler graphs. All are discussed further in STAR methods WGT’s graph generating algorithms, and in Figures S1 and S2. For graphs that start from biological sequences, we randomly selected 25 genes and downloaded their DNA and protein ortholog alignments in FASTA format from the Ensembl Comparative Genomics REST API^12^ at https://rest.ensembl.org/documentation/info/genomic_alignment_region. All the experiments are conducted on a 24-core, 48-thread Intel(R) Xeon(R) Gold 6248R Linux computer with 1024 GB memory, using a single thread of execution.

### Comparing Wheelie with Gibney & Thanckachan

Gibney and Thankachan’s recognition algorithm^9^ (henceforth “G & T”) works by enumerating all possible values for the *O*, *I*, and *L* arrays making up the Wheeler graph structure as described by Gagie et al.^11^ The *O* bitarray is a concatenation of unary codes describing the outdegrees of each node. *I* is a similar bitarray that does the same for indegrees. *L* is a sequence of characters labeling the edges in the order they appear in the *O* array. Further, the inner loop of the algorithm must check if a given assignment for *I*, *O*, and *L* is isomorphic to the input graph provided.

**Figure 1:**
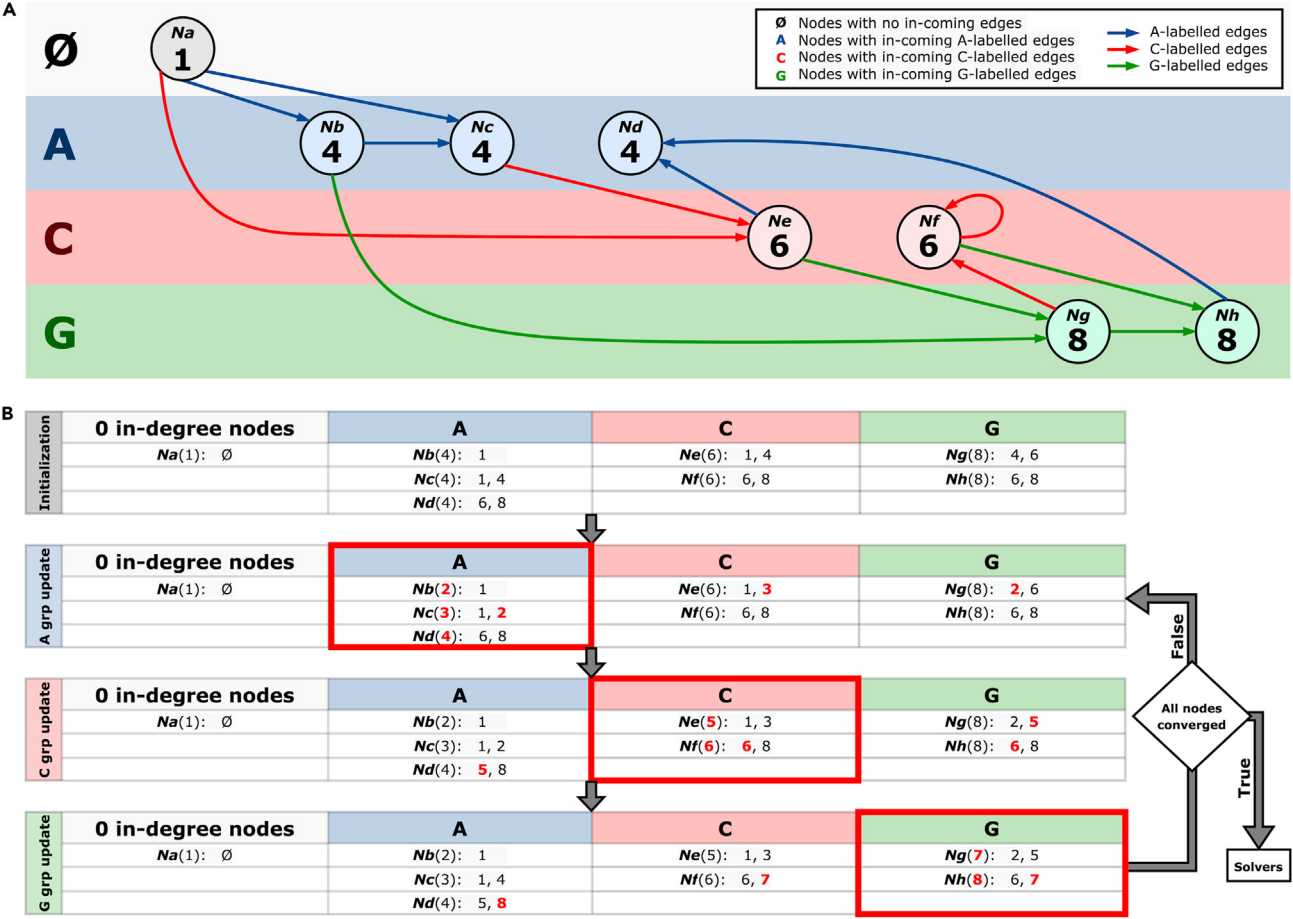
Illustration of the *renaming heuristic* (A) An 8-node graph with nodes divided into four groups according to in-coming edge label (with *O* representing 0-indegree nodes). (B) presents the workflow of the *renaming heuristic*. The first table in (B) shows the initialized in-node lists for eight nodes. After initialization, the algorithm sorts and relabels nodes in each group until convergence. Then, it passes the range information to either Wheelie-Pr or Wheelie-SMT.

While the G & T algorithm explores an exponential-sized space, Wheelie explores the factorial-sized space of node permutations. To consider how this affects the size of an exhaustive search, we compared the search space for the worst-case scenarios for both algorithms in Table S1. We hypothesized that Wheelie could be made faster with the help of strategies for pruning the search space. Wheelie prunes its search by assigning labels to nodes according to their *rough* positions in the order, a strategy we call the “*renaming heuristic*”. This allows Wheelie to arrive rapidly at a rough ordering that either (a) reveals a conflict that prevents the graph from having the Wheeler properties or (b) reduces the problem size for the downstream solving algorithm. An 8-node example illustrating the renaming heuristic is shown in Figure 1, and the full algorithm is described in STAR methods wheelie and the renaming heuristic, satisfiability modulo theories (SMT) solver. Here, we use a version of the algorithm called Wheelie-Pr, which begins with the *renaming heuristic* then resolves remaining ambiguities by exhaustively searching over the remaining node permutations. Unlike G & T, the Wheelie-Pr algorithm does not need to compute graph isomorphsims.

We conducted a 30-s timeout test on both algorithms using graphs generated from four generators (WGT’s graph generating algorithms) including both Wheeler and non-Wheeler graphs. Rather than implement G & T’s entire algorithm, we implemented the enumeration of the *I*, *O*, and *L* arrays but omitted the graph isomorphism check in the inner loop. We reasoned that if Wheelie-Pr was faster than the G & T algorithm without the (rather complex) isomorphism check, it would also be faster than the full G & T algorithm. To compare the algorithms, we configured both to perform an exhaustive search, without the possibility of early stopping if a solution is found. This differs from Wheelie-Pr’s default behavior, which allows it to stop upon finding a node ordering for which the Wheeler properties are satisfied. Early stopping is still possible for Wheelie-Pr in these experiments, since it might identify a conflict that proves the graph is non-Wheeler.

We took 25 multiple ortholog alignments, both their DNA and amino acid (AA) sequences, and extracted the first 4 rows of each. To reduce graph size, we truncated the graphs with respect to the multiple-alignment columns. We tested on three types of graphs, De Bruijn graphs, tries, and random Wheeler graphs, that are known Wheeler graphs, and two types of graphs, pseudo-De Bruijn graphs and reverse deterministic graphs, that are not guaranteed Wheeler graphs. Pseudo-De Bruijn graphs are graphs where the nodes correspond to k-1-mers in the multiple alignment, but where we do not collapse identical k-1-mers into single nodes.

**Figure 2:**
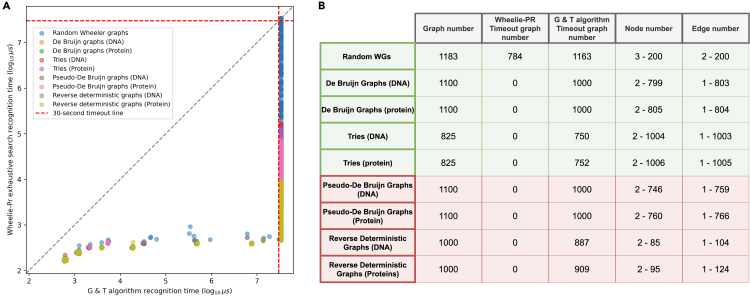
The results of comparing Wheelie-PR and G & T algorithm (A) Recognition time comparison between Wheelie-Pr exhaustive search and the G & T algorithm using (1) De Bruijn graphs, (2) tries, (3) pseudo-De Bruijn graphs and (4) reverse deterministic graphs generated from DNA and protein alignments, and (5) random graphs generated with given *n*, *e*, and σ. Wheelie-Pr recognition time is on the y axis, and G & T recognition time on the x axis, both on a $\log_{10}\text{microsecond}$ scale. Each dot represents a graph. Dots beyond the red lines denote inputs for which the tool timed out after 30 s. (B) The graph number, timeout graph number of Wheelie-Pr and the G & T algorithm, and ranges of node and edge number of each type of graphs. Rows in green describe graphs that are guaranteed Wheeler. Rows in red describe graphs that are not guaranteed to be Wheeler; i.e., some instances are Wheeler and some are not.

For De Bruijn graphs and pseudo-De Bruijn graphs, we took columns 1 to 200 and set *k* to 3 to 9; for tries, we took columns 1 to 200; for reverse deterministic graphs, we took columns 2 to 41. We also benchmarked with a series of randomly generated graphs with *n* set to 3 to 33, *e* from 3 to *n*, and σ from 1 to 21. The number of each type of graphs and their node and edge numbers are shown in Figure 2B, and the arguments of each generator can be found in the WGT Github repository: https://github.com/Kuanhao-Chao/Wheeler_Graph_Toolkit.

Figure 2A shows that Wheelie-Pr is significantly faster, allowing it to recognize a range of Wheeler and non-Wheeler graphs. Wheelie-Pr runtimes generally range from 100 to 1,000 μs, with 784 random-graph inputs causing Wheelie-Pr to time out. In sum, the only type of graph that caused Wheelie-Pr to time out is the random graph whereas 8,461 graphs distributed in all types of benchmarked graphs caused G & T to time out.

### Visualizing and characterizing challenging graphs

**Figure 3:**
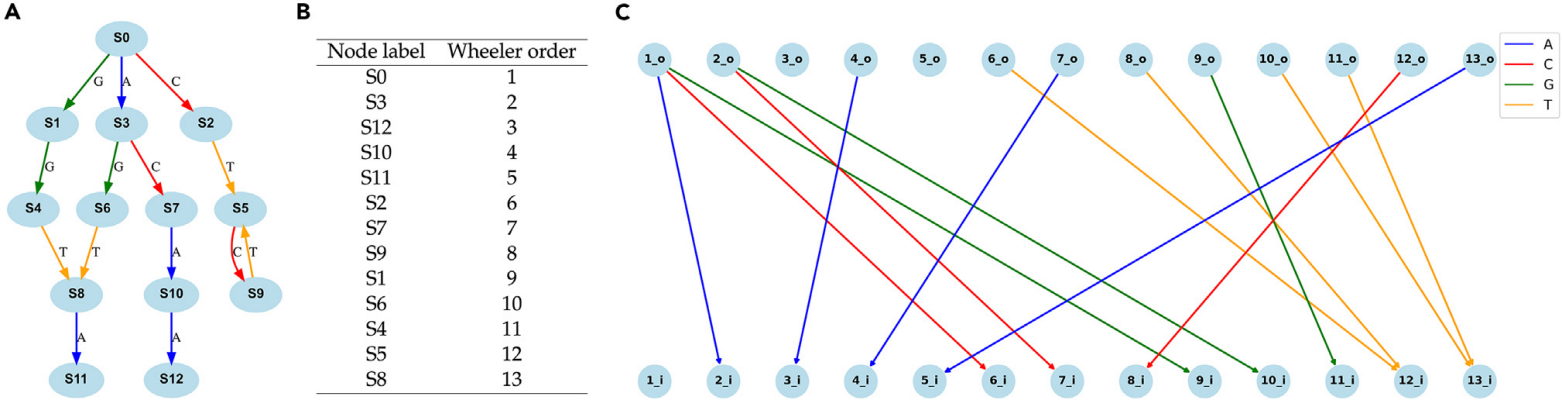
WGT recognition and visualization results of an *k=4* De Bruijn graph (A) A k=4 De Bruijn graph outputted from WGT’s De Bruijn graph generator. It is the visualization from Graphviz online visualizer. (B) The recognition result showing the Wheeler ordering outputted from Wheelie-Pr. (C) The output from WGT’s visualizer. Nodes are duplicated into two rows ordered in Wheeler ordering.

**Figure 4:**
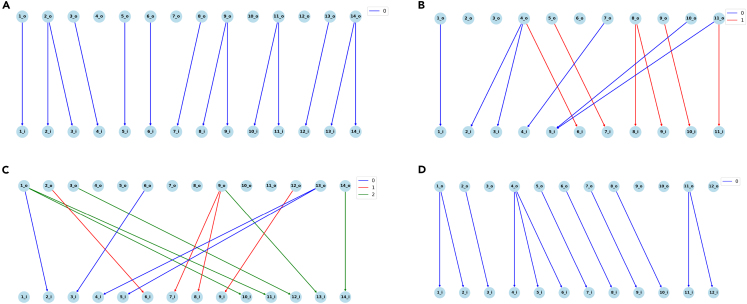
The results of WGT visualizer on d-NFAs with different numbers of labels Examples of (A) 2-NFA with 1 label (B) 2-NFA with 2 labels (C) 2-NFA with 3 labels (D) 3-NFA with 1 label, from outlier random graphs (blue dots) in Figure 2.

We selected a De Bruijn graph with edges being k-mers and nodes being k-1-mers where k=4 from the Figure 2 benchmarks. This graph was derived from the first four rows of the multiple alignment of STAU2 DNA orthologs with sequence length 4. We first visualized it using Graphviz^13^ (Figure 3A). We ran Wheelie-Pr to find an ordering for which the Wheeler properties hold (Figure 3B). Finally, we visualized the graph using WGT’s Python-based visualizer, which draws the ordered nodes in two replicas, with outgoing edges leaving one replica (Figure 4, top rows) and entering the other (bottom rows). For a valid Wheeler ordering, nodes with no incoming edges will appear leftmost, nodes with incoming edges of the smallest character will come next, nodes with incoming edges of the next-smallest character next, etc. Further, no two same-color edges will cross each other. In this way, the diagram, first described by Boucher et al.,^14^ makes it visually obvious when an ordering has yielded the Wheeler properties.

We sought to understand which graphs require the most time for recognition. After investigating the “outlier” graphs where Wheelie-PR timed out with these tools, we found that the graphs requiring the most recognition time tended to have nodes with many outgoing same-label edges. Following Alanko et al.,^15^ we use the term *d*-NFA to describe a Wheeler graph where all nodes have ≤d outgoing same-label edges, and at least one node has exactly *d* outgoing same-label edges. The De Bruijn graph shown in Figure 3 is a 1-NFA. Figures 4A–4C are 2-NFAs with σ equal to 1, 2, and 3, respectively. Figure 4D is a 3-NFA with σ=1. Note that the d≥5 case is the one proven to be NP-complete.^9^ De Bruijn graphs and tries are 1-NFAs.

#### Recognizing challenging graphs with Wheelie-SMT

Motivated by previous work that showed how Boolean satisfiability formulations can solve special cases of the recognition problem,^15^ we hypothesized that SMT solvers^10^ could solve all or part of the Wheeler graph recognition problem. SMT has found many uses in artificial intelligence and formal methods for hardware and software development. As a generalization of the Boolean satisfiability,^16^ SMT allows us to encode the Wheeler graph properties in a fairly straightforward way, building from the propositional logic formulas in the definition.

We conducted two series of 1,000-s timeout tests using graphs generated from the random generator comparing (1) Wheelie-Pr (*renaming heuristic* plus permutation) versus (2) Wheelie-SMT (*renaming heuristic* plus SMT) on different types of *d*-NFA (Recognizing *d*-NFAs) and various sizes of random graphs (Recognizing random Wheeler graphs).

### Recognizing *d*-NFAs

**Figure 5:**
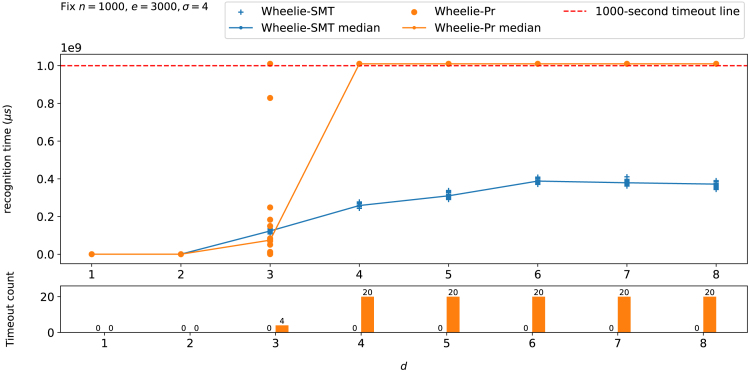
Recognition time for Wheelie-SMT and Wheelie-Pr as a function of the *d* parameter of the *d*-NFA Upper panel plots recognition time versus *d* and includes a line connecting the medians. 20 graphs were tested for each *d*. The bottom bar chart shows the number of timeouts.

We fixed n=1000, e=3000, and σ=4 and randomly generated *d*-NFAs with *d* from 1 to 8 and each group with 20 graphs. Figure 5 shows that both solvers can solve graphs swiftly when *d* is 1 and 2; as *d* grows beyond 2, all tools require much more time, demonstrating that *d* impacts the hardness of recognition problem in practice. Wheelie-SMT outperforms Wheelie-Pr and avoids any timeouts; Wheelie-Pr has some timeouts starting at d=3 (4 out of 20 graphs), and consistently times out when d≥4.

Further, we observed that when d≥6, the median curve for Wheelie-SMT plateaus. This is because *n* and *e* are too small for the *d*-NFA generator to produce uniformly distributed *d*-NFAs under the given parameters. More precisely speaking, the hardness of the recognition problem is a function of the distribution of nodes having d−1, d−2, …, 1 outgoing edges with the same labels. As an example, take a *d*-NFA *G* that has one node with *d* same-label outgoing edges, and the rest of the nodes having at most one outgoing same-label edge. Recognizing *G* is not harder than recognizing a uniformly distributed d−1-NFA. In short, we observed that higher *d*s generally led to a harder recognition problem, but the true level of hardness was also a function of *n*, *e*, and σ.

### Recognizing random Wheeler graphs

**Figure 6:**
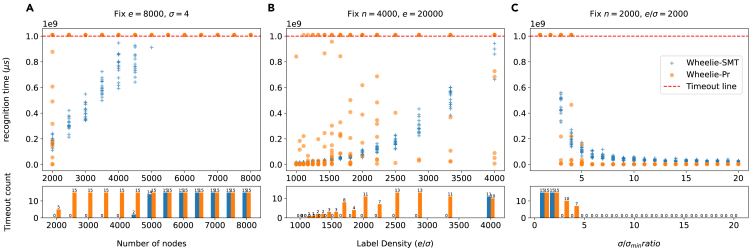
Recognition time comparison for Wheelie-SMT and Wheelie-Pr for various random Wheeler graphs Three experiments were conducted. (A) Experiment 1: fixing e=8,000, σ=4 and scaling up the graph size (*n* from 2,000 to 8,000). (B) Experiment 2: fixing n=4,000, e=20,000 and scaling up the label density (e/σ from 1,000 to 4,000). (C) Experiment 3: Fixing both graph size (*n*) and label density (e/σ), and scaling up both *e* and σ ($\sigma /\sigma_{\min}$ from 1 to 20). The upper-panel plots show the recognition time versus the scale up parameter in microsecond scale. Each dot represents a graph. Dots beyond the red dashed line means timeouts. Plots in the lower panel are the timeout count bar charts.

We defined “graph size” as *n* and “label density” as e/σ. We then benchmarked various sizes of random graphs while varying these parameters. We first fixed the number of edges (e=8,000) and labels (σ=4) while scaling graph size *n* from 2,000 to 8,000. Figure 6A shows that as *n* grows, Wheelie-SMT outperforms Wheelie-Pr significantly. Wheelie-Pr starts to time out in some cases when n=2,000, and most cases when n≥2,500. In contrast, Wheelie-SMT can solve all cases with *n* up to 4,000, and most cases when n=4,500.

We then fixed the graph size (n=4,000) and number of edges (e=20,000) and varied the label density (e/σ from 1,000 to 4,000). Figure 6B shows that as the label density increases, the graphs take more time to solve. Comparing Wheelie-Pr and Wheelie-SMT, we can see that there are more timeout cases in Wheelie-Pr from 1,200 to 4,000 (most are timeouts when e/σ≥2,500) whereas the timeout cases only occur in Wheelie-SMT when label density is 4,000.

In a third experiment, we fixed the graph size (n=2,000) and varied the number of edges (*e*) and labels (σ) while fixing the label density ratio (e/σ=2,000). Figure 6C shows that as more edges and labels are added, the recognition problem becomes easier. In short, this is because adding more constraints to *G* breaks more of the ties that would otherwise obstruct Wheelie’s *renaming heuristic*. Comparing Wheelie-Pr to Wheelie-SMT, Figure 6C shows that the solvers perform similarly, with Wheelie-Pr performing slightly better when $\sigma /\sigma_{\min}$ ratio gets larger (≥5). These are likely cases where the graph is sufficiently easy to recognize that the overhead of setting up the SMT setup problem becomes harmful. When $\sigma /\sigma_{\min}$ gets smaller (3 and 4), Wheelie-SMT is able to solve all 15 cases, whereas Wheelie-Pr’s times out for about half the cases.

### Benchmarking Wheelie-SMT alone

To isolate the effect of the Wheelie *renaming heuristic*, we conducted a 30-s timeout test with 60 s timeout penalties on (1) Wheelie-SMT (*renaming heuristic* plus SMT) and (2) a pure SMT solver starting from scratch, without the constraints it would otherwise receive from the *renaming heuristic*. We benchmarked these using two generators from DNA alignments: (1) De Bruijn graphs generated with options -k from 5 to 8, -l from 100 to 2,000, and -a from 6 to 10, 225 graphs in total and (2) reverse deterministic graphs generated with options -l from 100 to 500 and -a from 4 to 6, in total 225 graphs.

**Figure 7:**
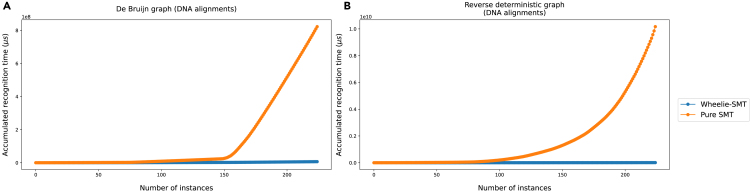
Cactus survival plot results of Wheelie-SMT and pure SMT (A and B) The cactus survival plots of (A) De Bruijn graphs and (B) reverse deterministic graphs generated from DNA alignments using WGT’s generators. They show the aggregated time comparison between Wheelie-SMT and pure SMT without the constraints from the *renaming heuristic*.

Figure 7 shows cactus plots on De Bruijn graphs and reverse deterministic graphs. A cactus plot is an aggregated sorted time plot widely used in solver competitions. It shows how many problems a solver can solve in a limited time period. In Figure 7A, Wheelie-SMT solved the whole De Bruijn graph set in around 6.5 s whereas the pure SMT approach solved it in around 820 s. For reverse deterministic graphs (Figure 7B), Wheelie-SMT solved the whole set in less than 9 s whereas the pure SMT approach solved it in around 10,170 s.

We concluded that the *renaming heuristic* is a crucial step, since it greatly narrows the space of possible node ordering that must be resolved by the SMT solver. Wheelie-SMT can solve graphs several orders of magnitude larger than a pure SMT approach.

## Discussion

We demonstrated that Wheelie-SMT is the fastest and most robust algorithm available for the Wheeler graph recognition problem. We showed this across a variety of graph types, including large graphs (thousands of nodes and edges) and challenging graphs, such as those that are *d*-NFAs with values of *d* up to 8. We also demonstrated WGT’s facilities for visualizing and understanding these graphs.

While current pangenome representations tend to be based on De Bruijn graphs, which are Wheeler graphs, other relevant pangenome graph representations are not necessarily Wheeler. For example, the reverse deterministic automata of the GCSA study^6^ are not Wheeler, though they can be made Wheeler through a “path doubling” process. In the future, we expect that WGT and the Wheelie algorithm will be useful for studying alternative pangenome graph representations that might improve upon De Bruijn graphs in various ways. In Figure S3, we provide a simple illustration of why another Wheeler graph (besides a De Bruijn graph) might be better suited as a pangenome representation, since it (a) uses fewer nodes and edges than a corresponding De Bruijn graph, (b) does not require that we select a particular value for *k* (the *k*-mer length), and (c) avoids collapsing sequences that are distinct with respect to the coordinate system of a given genome.

We noted a relationship between the renaming heuristic proposed here and the “forward algorithm” of Alanko et al.^17^ In the future, it will be important to clarify the relationship between these two algorithms, which have similar goals but take different approaches to partitioning and ordering the graph nodes. Appealingly, the forward algorithm has polynomial running time when the input is already a Wheeler graph; we do not have a similar guarantee for the renaming heuristic. However, the forward algorithm is not directly usable for the same purpose as the renaming heuristic, since it is possible for the forward algorithm to collapse a non-Wheeler input in a way that produces a Wheeler output. Another question for future work is whether the renaming heuristic could be combined with the forward algorithm to obtain an algorithm with strong guarantees (like the forward algorithm) but that is directly applicable to the recognition problem (like the renaming heuristic).

When Wheelie determines that a graph is is Wheeler graph, it is able to report a node ordering that can then be used to index the graph. In the future, it will be important to extend Wheelie to report other useful information, including when the graph is not a Wheeler graph. For instance, when Wheelie encounters a conflict that proves the graph to be non-Wheeler, Wheelie could supply the user with an explanation for why the graph cannot be Wheeler. Such an explanation could also allow Wheelie to suggest modifications to the graph that would make it a Wheeler graph, without changing which strings it encodes. A trivial example would be a node with two incoming edges having two distinct labels. This violates the Wheeler graph properties, but also suggests a potential solution: the node could be duplicated, with outgoing edges also duplicated. The initial inbound edges could be redrawn to point to the distinct duplicates, possibly restoring the Wheeler properties. A more general approach for understanding Wheeler violations could work by extracting conflicting sets of clauses from the SMT algorithm, and converting them into a human-understandable or other actionable form.

It may also be possible to encode the *renaming heuristic* as a set of clauses in the SMT solver, potentially allowing the entire algorithm to execute within the SMT solver. Finally, as different SMT solvers such as CVC5^18^ or Z3^19^ adopt different heuristics, they could potentially be substituted into WGT, or combined for increased efficiency.^20^

### Limitations of the study

A theoretical limitation is that we cannot claim to have improved on the worst-case bounds already established for the recognition problem. The problem is NP-complete in general, though our work suggests that the kinds of graphs that appear in pangenome applications (e.g., when they are *d*-NFAs with small *d*) may be of a class that are easier to check in practice. A further practical limitation is the fact that Wheelie cannot yet scale to large pangenome graphs. Wheelie has been demonstrated to effectively identify graphs with up to 4,000 nodes, 20,000 edges, and 5 edge labels within a time frame of around 1000 s. We are still far from being able to efficiently check pangenomes derived from, for example, whole eukaryotic genomes. A final limitation is the fact that our bipartite visualization approach works only when a particular ordering has been proposed. A more general approach would render a useful visualization when such an ordering is either not known or partially known.

## STAR★Methods

### Key resources table

**Table:** 

REAGENT or RESOURCE	SOURCE	IDENTIFIER
**Deposited data**		

25 multiple orthologue alignments	Ensembl Comparative Genomics REST API	REST API: https://rest.ensembl.org/documentation/info/genomic_alignment_region; GitHub: https://github.com/Kuanhao-Chao/Wheeler_Graph_Toolkit/tree/main/data/multiseq_alignment/Ensembl_REST/fasta

**Software and algorithms**		

G & T’s algorithm	Gibney & Thankachan	https://doi.org/10.48550/arXiv.1902.01960
Z3 theorem prover v4.11.1	Microsoft Corporation	https://github.com/Z3Prover/z3
WGT toolkit v1.0.0	This study	Zenodo DOI: https://doi.org/10.5281/zenodo.7937689; GitHub: https://github.com/Kuanhao-Chao/Wheeler_Graph_Toolkit

### Resource availability

#### Lead contact

Further information and requests for resources and reagents should be directed to and will be fulfilled by the lead contact, Ben Langmead (langmea@cs.jhu.edu).

#### Materials availability

This study did not generate new unique reagents.

### Method details

#### The search space of Wheelie’s permutation approach

While the G & T algorithm explores an exponential-sized space of possible array assignments, Wheelie explores a factorial-sized space of node permutations. This may or may not lead to a larger search space for Wheelie, depending on the graph’s properties. To be specific, the G & T’s algorithm may have to consider all $2^{2\left(e+n\right)+e\;\log\left(\sigma \right)}$ assignments for *I*, *O* and *L*. Our approach might need to consider n! node permutations in the worst case. We sought a rough comparison between the approaches in light of the fact that G & T’s space depends not only on *n* but also on *e* and σ.

(Equation 1) $$2^{2\left(n+e\right)+e\times \log\;\sigma }=n!$$

(Equation 2) $$2^{2n+e\times \left(2+\log\;\sigma \right)}=n!$$

LetC:=e(2+logσ)

(Equation 3) $$2n+C=\log_{2}\;n!$$

(Equation 4) $$C=\sum_{x=1}^{n}\log_{2}\;x-2n$$

#### Derivation 1: The relationship between *C* and *n*

In Derivation 1, we fixed *n* for both, defining a new variable *C* as e(2+logσ). We then found some values for *C* that equalize the algorithms’ search space size under various values for of *n*s (Table S1). For instance, when n=100, *C* can be at most 324 in order for G & T’s algorithm has an equal or smaller search space than Wheelie-Pr, which is a strict threshold, and furthermore, this comparison is done with Wheelie-Pr skipping the *renaming heuristic*, which in reality makes Wheelie-Pr superiorly faster (ResultsBenchmarking Wheelie-SMT alone).

To gain a further advantage over the G & T algorithm, Wheelie further strives to prune the search space, using a renaming heuristic, an SMT solver, or both, as detailed in MethodsSTAR Methods.

#### Wheelie and the *renaming heuristic*

Wheelie explores the space of possible node orderings until arriving either at a conflict (e.g., a node with distinctly labeled incoming edges) or an ordering for which the Wheeler properties hold. While this is a large (n!-sized) search space, Wheelie prunes the space by assigning labels to nodes according to their *rough* position in the overall order. Initially, a rough ordering is determined according to the labels of the immediate incoming edges for each node, following the Wheeler requirement that $a≺a^{\prime }\to v<v^{\prime }$ for all edge pairs. This rough ordering is refined over the course of a procedure that iterates either until the rough ordering becomes total ordering, or until the rough ordering stabilizes. In the latter case, the remaining ambiguities are resolved by a non-heuristic solver. This procedure is detailed in Algorithm 1 and illustrated in Figure 1.

Algorithm 1 **Wheeler graph Recognition Algorithm, Wheelie**

**Require**:

**Input:** Graph *G* as DOT file.

**Input:** Solver *S* input from -s or --solver tag.

1: Find all 0-indegree nodes, Roots[]
2: Group edges by their labels in a hashMap, label_2_edges (key: label, value: list of edges)
3: accum_order←Roots.size() ▷ Relabel nodes with the largest possible order
4: **for** each root∈Roots **do**
5:  relabel_node(root,accum_label)
6: **end for**.
7: **for** each [label,edges]∈label_2_edges **do**
8:  $accum\_order\leftarrow accum\_order+unique\left(edges.destination_{nodes}\right).size\left(\right)$
9:  **for** each edge∈edges **do**
10:  relabel_node(edge.destination_node,accum_order)
11:  **end for**.
12: **end for**.
13: converged←False ▷*renaming heuristic*
14: ranges[]
15: **while** not converged **do**
16:  ranges.clear()
17:  accum_order←0
18:  innode_list[]
19:  **for** each [label,edges]∈label_2_edges **do**
20:  nodes←unique(edges.destination_nodes)
21:  **for** each node∈nodes **do**
22:  innode_list.Add(get_innodelist(node)) ▷ get_innodelist: lists distinct predecessor nodes in order by label
23:  **end for**.
24:  indices=sort_node_by_innodelist(nodes,innode_list)
25:  relabel_node(nodes,innode_list,indices,accum_order) ▷ Assign order_range to prev_order_range for each node
26:  accum_order←accum_order+nodes.size()
27:  **end for**.
28:  converged←True
29:  **for** each node∈nodes **do**
30:  ranges.Add(node.order_range)
31:  **if**node.order_range≠node.prev_order_range**then**
32:  converged←False
33:  Break
34:  **end if**.
35:  **end for**.
36: **end while**.
37: **if**unique(ranges).size()=ranges.size()**then** ▷ Solved by *renaming heuristic*
38:  *G* is a Wheeler graph.
39: **else**.
40:  **if**S=='' Permutation'' **then**
41:  Permutation(G,ranges) ▷ Use Wheelie-Pr solver to resolve multi-node groups
42:  **else if**S=='' SMT'' **then**
43:  SMT(G,ranges) ▷ Use Wheelie-SMT solver to resolve multi-node groups
44:  **end if**.
45: **end if**.

As the *renaming heuristic* iterates, it repeatedly visits the nodes in groupings according to the label of their incoming edge(s). For each of these groupings, it sorts the edges by sources and destinations in every label group, requiring $O\left(\prod_{g\in \Sigma }e\left(g\right)\log_{2}\;e\left(g\right)\right)$ time, where e(g) is the number of edges labeled as *g*. We observed that many non-Wheeler graphs can be recognized as such directly by the *renaming heuristic*, without requiring a downstream solver.

At each iteration, the algorithm gathers a list of sorted unique temporary orders of nodes that go into it, which we term the “in-node list.” By the Wheeler graph property that requires all edge pairs to satisfy $a=a^{\prime }\wedge u<u^{\prime }\to v\leq v^{\prime }$, we can find rough orders by sorting the nodes by their in-node lists. Once this has been done for each node group, we reach the end of the current iteration and we check if the rough order changed since the previous iteration. If not, then we say the algorithm has converged and forward any remaining ambiguities to the downstream solver as necessary.

We note that there are similarities between the renaming heuristic and the “forward algorithm” of Alanko et al.^17^ (Algorithm 2 in that paper). While our renaming heuristic performs an explicit sort within each of its rough grouping, the forward algorithm of Alanko et al. uses a pair of nested loops over alphabet characters to visit and partition the nodes in a way that maintains their sorted order implicitly. We discuss this relationship further in Discussion Discussion below.

##### Wheelie contains two solvers

Wheelie-PR and Wheelie-SMT. Wheelie-PR takes the output from the renaming heuristic and, for any remaining ties in the ordering, simply tries all possible permutations among the tied nodes. The Wheelie-SMT solver is explained in the next subsection Satisfiability Modulo Theories (SMT) solver.

#### Satisfiability Modulo Theories (SMT) solver

Motivated by the use of boolean satisfiability formulations to solve special cases of the recognition problem,^15^ we hypothesized that Satisfiability Modulo Theory (SMT) solvers^10^ could be used to solve all or part of the Wheeler-graph recognition problem. SMT has found many uses in artificial intelligence and formal methods for hardware and software development. As a generalization of the Boolean Satisfiability (SAT),^16^ SMT allows us to encode the Wheeler graph properties in a fairly straightforward way, building from the propositional logic formulas in the definition.

An SMT problem decides the satisfiability of a first-order formula with respect to one or more background theories. A *formula* is a set of atoms connected by Boolean connectives (∧, ∨, ¬), where an *atom* is a predicate which valuates to True or False given an assignment to the variables. A *literal* is either an atom or its negation. A *theory* gives special meanings, known as *interpretations*, to functions and predicate symbols within the theory. In this paper, we consider only the theory of Integer Difference Logic (IDL), which requires atoms to be of the form $x_{i}-x_{j}\leq c$, where $x_{i}$ and $x_{j}$ are integer variables, *c* an integer constant, “−” the integer subtraction function, and “≤” the usual binary ordering predicate. A theory solver decides the satisfiability of a conjunction of *literals*. In particular, an IDL theory solver can be implemented as the Bellman-Ford algorithm which runs in polynomial time. Incorporating a SAT solver and a theory solver, an SMT solver takes in a formula and outputs an assignment to the variables if the formula is satisfiable or otherwise reports unsatisfiability.

We observed that SMT is a natural way of encoding the Wheeler graph recognition problem. Firstly, for each node a variable is created representing the ordering of the node. Recalling the constraints, for all pairs of edges (u,v) and $\left(u^{\prime },v^{\prime }\right)$ labeled *a* and $a^{\prime }$ respectively: (i) $a≺a^{\prime }\to v<v^{\prime }$, and (ii) $a=a^{\prime }\wedge u<u^{\prime }\to v\leq v^{\prime }$. By indexing the known labels lexicographically, we obtained an SMT formula containing constraints (i) and (ii), in which all atoms are of the form $v\leq v^{\prime }$ satisfying the IDL requirement. Note that the strict inequality $v<v^{\prime }$ can be rewritten using the the non-strict one as $v-v^{\prime }\leq -1$. Besides constraints (i) and (ii), we also enforced all-different constraints and range constraints for all nodes, which have the form 1≤v≤n. The node orderings can be obtained from the satisfying assignment by solving the SMT formula iff it is satisfiable. In Wheelie-SMT, we used Z3^19^ as the underlying SMT solver.

As the number of constraints and variables are the main factors affecting runtime, simplifying the problem or providing additional information can improve performance. We noticed that the problem can be simplified using the rough order obtained from the *renaming heuristic* described in Section wheelie and the renaming heuristic. By adding the range information to the formula, notice that constraint (i) can be removed from the formula. Moreover, the all-different constraints of nodes from different groups can also be removed. The rationale is that if the graph was not reported non-Wheeler during the *renaming heuristic*, the range constraints provided by the procedure must automatically imply constraint (i). The benefits are 2-fold: not only the number of constraints is reduced, the search space is also significantly pruned.

#### WGT’s graph generating algorithms

We implemented five generators in Python scripts to produce tries, reverse deterministic graphs,^6^ De Bruijn graphs and *complete* and *d-NFA* random Wheeler graphs. The first three generators take either DNA or protein multiple sequence alignments and produce the corresponding graph structures. As for the two random generators, users can produce a Wheeler graph given its *n*, *e*, and σ, and *d-NFA* random generator can further take *d*, which controls the most number of edges coming out from a node with the same label (*d*-NFA) as user input.

Tries and De Bruijn graphs are Wheeler graphs by definition. These generators start by removing gap placeholders from the multiple alignments. The trie generator iterates through the prefixes of each sequence in the multiple alignment, inserts characters into the trie and creates a new node at the end of a path if a prefix cannot be traversed from the source. Edges are labeled according to the label of the parent. The De Bruijn graph generator constructs a distinct k−1-mer dictionary from the sequences. It connects edges between adjacent two nodes and label the edge with the first character in the k−1-mer of the child node. Reverse deterministic graphs are usually invalid Wheeler graphs but might be valid when the graphs are small, and once violations occur, adding more nodes and edges cannot turn them back to Wheeler graphs.

The reverse deterministic graph generator iterates through columns of a multiple sequence alignments from right to left. At a column *i*, it creates distinct nodes for the characters found there, connecting them to the current node with the node of the previous ungapped character with the direction pointing to the end of the alignments and the label of the previous ungapped character. This follows the procedure described in the GCSA study.^6^ Last, three generators initializes the names of nodes with the breadth first search orders and outputs the constructed graph in DOT format.

We also implemented two random generators, a *complete* Wheeler graph generator and a *d-NFA* Wheeler graph generator. We first fix the ordering of nodes and then try to select edges such that both user-specified constraints and Wheeler graph properties are satisfied. Let $N_{i}$ be the nodes with incoming edges labeled *i* and $E_{i}$ be the edges labeled *i* where i=1,2,…,σ, and also let $n_{i}=\left|N_{i}\right|$ and $e_{i}=\left|E_{i}\right|$. In both generated graphs, we assume that $n_{i}\approx \frac{n-r}{\sigma }$ and $e_{i}\approx \frac{e}{\sigma }$ where *r* is the number of nodes without incoming edges.

We say a Wheeler graph *G* is *complete* if no more edges can be added to *G* while maintaining the Wheeler graph properties.

Property 1. Given number of nodes n and number of labels σ, the number of edges of a Wheeler graph is upper bounded by $e_{\max}$ *where*

(Equation 5) $$e_{\max}=n\times \sigma +n-\sigma -r=\left(n-1\right)\left(\sigma +1\right)-r+1$$

Proof. Consider the bipartite representation of a Wheeler graph *G* with number of nodes *n* and number of labels σ. Note that

(Equation 6) $$\sum_{i=1}^{\sigma }n_{i}=n-r\text{.}$$

Observe that for each label *i*, the number of edges that is labeled *i* is at most $n+n_{i}-1$. Taking the sum of edges of each label and applying Equation 6, we have

(Equation 7) $$e_{\max}=\sum_{i=1}^{\sigma }\left(n+n_{i}-1\right)=n\times \sigma -\sigma +\sum_{i=1}^{\sigma }n_{i}=n\times \sigma -\sigma +n-r\text{.}$$

One way of generating complete Wheeler graphs is to have all nodes connect to the first node of $N_{i}$ and the last node additionally connect to the rest of the nodes in $N_{i}$ for each label *i* (the last node has $n_{i}$ outgoing edges in total). By randomly selecting $n_{i}-1$ nodes from *N* and connecting the selected nodes to consecutive nodes in $N_{i}$, a new complete Wheeler graph can be generated by appropriately shifting the destination node of each edge such that the Wheeler graph property is maintained. Figure S2 shows an example of a complete Wheeler graph with (n,e,σ,r)=(7,18,2,1). With a complete Wheeler graph of *n* nodes and σ labels, we are able to generate random Wheeler graphs with $e<e_{\max}$ edges by sampling *e* distinct edges from the complete Wheeler graph.

For generation of *d*-NFA Wheeler graphs, let $x_{k}$ be the number of nodes with *k* outgoing edges of the same label *i*. Thus, given *e* and σ we have

(Equation 8) $$\sum_{k=1}^{d}k\cdot x_{k}=e_{i}$$

Also the number of nodes $n_{i}$ with incoming edges labeled *i* must be greater than the minimum number of nodes $n_{\min}$ needed to accommodate $e_{i}$ edges. Thus, we have

(Equation 9) $$n_{\min}=1+\sum_{k=2}^{d}\left(k-1\right)\cdot x_{k}\leq n_{i}$$

Note that any solution $x_{k}$ that satisfies Equations 8 and 9 for all edge label *i* represents a set of valid Wheeler graphs. To see this first notice that given $x_{k}$, we can always order the nodes such that $x_{1}$ nodes with one outgoing edge are placed at the front, followed by $x_{2}$ nodes with two outgoing edges, followed by $x_{3}$ nodes with three outgoing edges and so on. By construction, this gives a valid *d*-NFA Wheeler graph. Moreover, by swapping the nodes and reconnecting the corresponding edges accordingly, different *d*-NFA Wheeler graphs can be obtained. An example is shown in Figure S3.

To obtain a concrete instance, we find a valid solution for $x_{k}$ and then determine the node ordering. In our case, we set all $x_{k}$ to be the same, and if not possible assign the residual to $x_{1}$ to satisfy Equation 8. We believe that this reflects the hardness of different benchmarks with different d’s. For node ordering, all the nodes are shuffled and edges are distributed such that each node in $N_{i}$ has at least one incoming edge while maintaining the Wheeler properties.

## Data Availability

• All original code has been deposited at Zenodo and is publicly available as of the date of publication. DOIs are listed in the key resources table. • The experimental datasets are listed in the key resources table. • WGT is publicly available at https://github.com/Kuanhao-Chao/Wheeler_Graph_Toolkit.
