# Supplementary material for: WGT: Tools and algorithms for recognizing, visualizing, and generating Wheeler graphs
Source: iScience. 2023 Jul 14;26(8):107402. doi: 10.1016/j.isci.2023.107402 (PMC10415921; doi:10.1016/j.isci.2023.107402)
Supplement: Document S1. Figures S1–S3 and Table S1 [file mmc1.pdf]

iScience, Volume *26*

Supplemental information

WGT: Tools and algorithms for recognizing, visualizing, and generating Wheeler graphs

Kuan-Hao Chao, Pei-Wei Chen, Sanjit A. Seshia, and Ben Langmead

n	C threshold
10	1.79
20	21.08
30	47.71
40	79.16
60	152.13
80	234.83
100	324.76
150	572.86
200	845.38

Table S1: Comparing the search space between `Wheelie`'s permutation approach and G & T's approach, related to STAR Methods "The search space of `Wheelie`'s permutation approach." Threshold values of C as a function of n such that values greater than the threshold cause the permutation-based approach to have a smaller search space compared to the G & T approach.

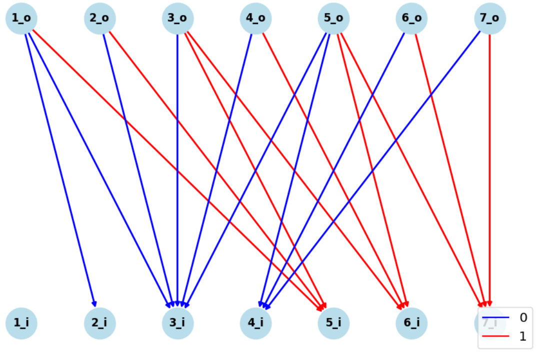

Figure S1: An example of a complete Wheeler graph, related to STAR Methods "WGT's graph generating algorithms" section. A complete Wheeler graph with $(n, e, \sigma, r) = (7, 18, 2, 1)$. In this example we have $n_0 = n_1 = 3$. The selected nodes for label 0 is node 1 and 5 and for label 1 node 3 and 5.

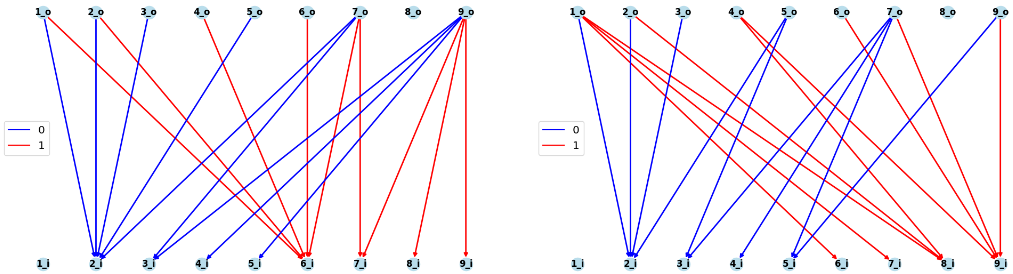

Figure S2: d-NFA generator, related to STAR Methods "WGT's graph generating algorithms" section. A 3-NFA Wheeler graph with $(n, e, \sigma, r) = (9, 18, 2, 1)$. On the left shows the original Wheeler graph and on the right shows the Wheeler graph after swapping nodes and corresponding edges. Our d-NFA generator directly generates Wheeler graphs with swapped nodes and edges.

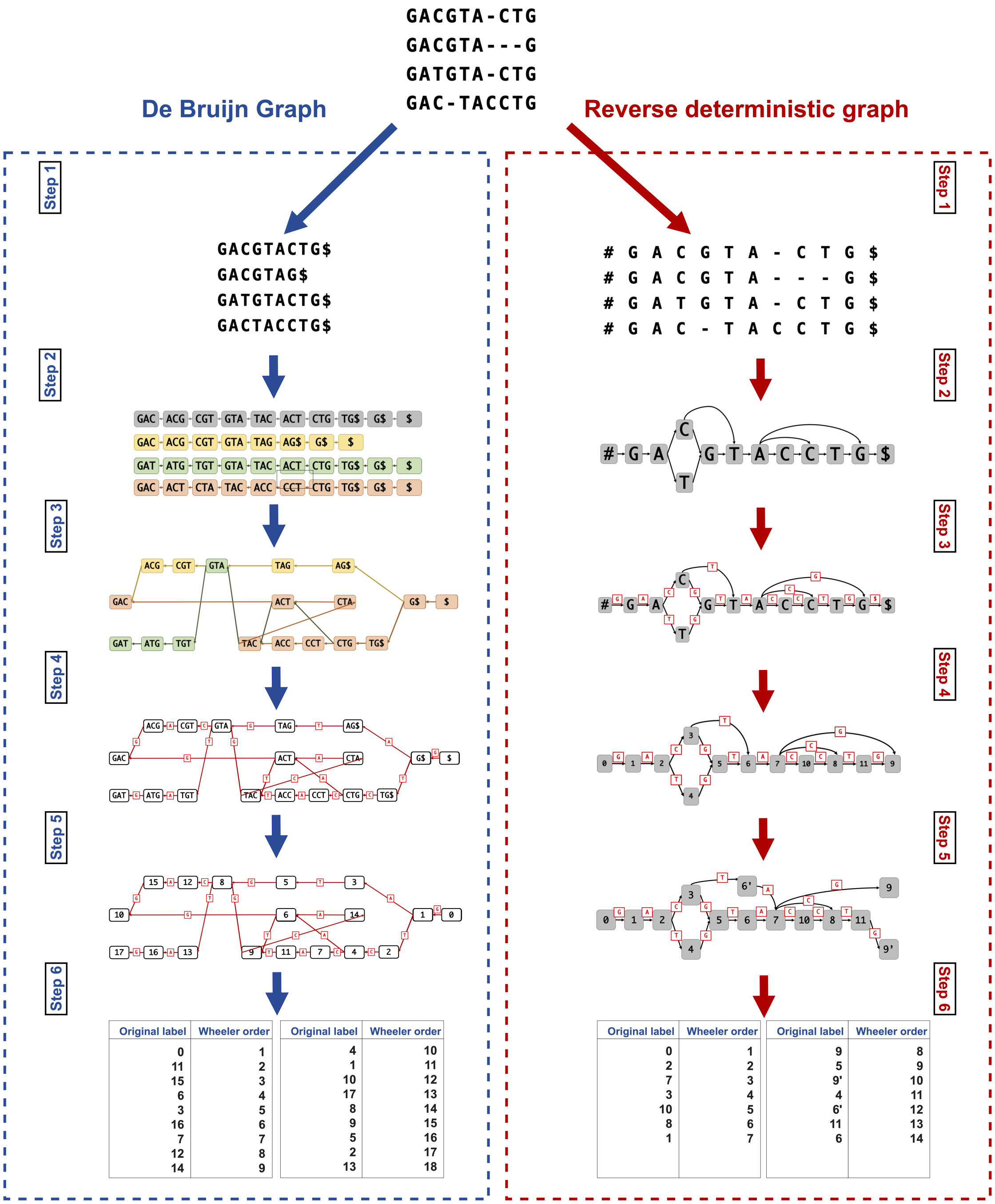

Original label	Wheeler order
0	1
11	2
15	3
6	4
3	5
16	6
7	7
12	8
14	9

Original label	Wheeler order
4	10
1	11
10	12
17	13
8	14
9	15
5	16
2	17
13	18

Original label	Wheeler order
0	1
2	2
7	3
3	4
10	5
8	6
1	7

Original label	Wheeler order
9	8
5	9
9'	10
4	11
6'	12
11	13
6	14

Figure S3: the benefit of recognizing Wheeler graphs from reverse deterministic graphs, related to Discussion. An example of creating a De Bruijn graph and a reverse-deterministic graph from a multiple sequence alignment. (1) The workflow on the left-hand side shows the process of creating a $k = 4$ De Bruijn graph. In the first step, gaps in the multiple sequence alignment are removed, and sequences are chopped into 3-mers (step 2). In step 3, nodes with the same 3-mers are merged, and the first character of each k-mer node is labelled on the edge that goes into it (step 4). In step 5, we do breadth-first search to relabel k-mer nodes. Finally, a De Bruijn graph with 18 nodes and 21 edges is created. (2) The workflow on the right-hand side shows the process of creating a reverse deterministic graph. In Step 1, we add '# at the beginning and '$ at the end of sequences. Step 2 involves merging nodes in each column that share the same sequences. These merged nodes are labeled with the node where the edge goes into, as shown in Step 3. Next, we relabel the nodes using breadth-first search in Step 4. If the current graph is not a Wheeler graph, as checked in Step 5, we unzip the nodes into bubbles. For instance, in this example, node 9 can be unzipped into node 9 and node 9′, and node 6 can be unzipped into node 6 and node 6′, resulting in a Wheeler graph with 14 nodes and 16 edges. Finally, we run our `Wheelie-PR` to index both the De Bruijn Graph and the reverse deterministic graph in step 6.
